# Primary endocervical gastric-type adenocarcinoma: a clinicopathologic and immunohistochemical analysis of 23 cases

**DOI:** 10.1186/s13000-019-0852-y

**Published:** 2019-07-06

**Authors:** Shanshan Lu, Danhua Shen, Yun Zhao, Nan Kang, Xingxing Wang

**Affiliations:** 1 Department of Pathology, Peking University People’s Hospital, 11Xizhimen South Street, Xicheng District, Beijing, 100044 China; 2 Department of Obstetrics and Gynecology, Peking University People’s Hospital, 11Xizhimen South Street, Xicheng District, Beijing, 100044 China

**Keywords:** Cervix, Gastric- type adenocarcinoma, Cytology, Histopathology, Immunohistochemistry, Differential diagnosis

## Abstract

**Background:**

Endocervical gastric-type adenocarcinoma (GAS) is a rare non-human papillomavirus-associated adenocarcinoma (NHPVA) with morphologic and immunohistochemical features of gastric differentiation. This study aimed to evaluate cytologic and clinicopathological features, differential diagnosis of endocervical GAS.

**Methods:**

A total of 23 patients diagnosed with endocervical GAS/minimal deviation adenocarcinoma (MDA) at Peking University People’s Hospital between 2009 and 2018 were included. Clinical characteristics, cytologic/histopathologic findings, and immunohistochemical results were collected and analyzed.

**Results:**

The average age of patients was 51 years old (range from 28 to 73). Cytologically, tall columnar epithelial cells with pale, foamy or vacuolated cytoplasm were mostly common, followed by well-defined cytoplasmic borders. Fourteen endocervical GAS cases demonstrated mild cytologic atypia, and 9 cases showed moderate to marked cytologic atypia. Ovarian and fallopian tube involvement were identified in 5 and 6 cases, respectively. Immunohistochemically, tumor cells were diffusely positive for CK7, MUC6 and CA-IX, but focally positive for CK20 and CDX2. P16 was negative or patchy positive in most cases and p53 mutation was identified in 12 cases (12/21, 57.1%).

**Conclusions:**

Endocervical GAS shows different morphologic and immunological features from endocervical usual type adenocarcinoma, but it may be difficult to be differentiated from metastatic mucinous adenocarcinoma to cervix due to similar morphology and overlapping immunohistochemical profile. Therefore, awareness of the morphologic features and immunohistochemical profile of GAS will allow pathologists to recognize and accurately diagnose this rare and aggressive entity.

## Background

More than 90% of cervical adenocarcinomas are caused by high-risk human papillomavirus (HPV) persistent infection [1, 2], with the most common HPV types as 18, 16 or 45 [3]. However, the remaining 5–10% of cervical adenocarcinomas are not associated with HPV infection, and the most common one is gastric-type adenocarcinoma (GAS). GAS is defined as a subtype of mucinous adenocarcinoma with gastric differentiation in the 2014 World Health Organization classification of cervical tumors. Minimal deviation adenocarcinoma (MDA), also known as adenoma malignum, is a designation that refers to a well-differentiated form of GAS. The histologic features of GAS include clear and pale eosinophilic cytoplasm, distinct cell borders, and immunohistochemical evidence of gastric differentiation (expression of MUC6 and HIK1083) [4].

Well differentiated GAS, such as MDA, is characterized with bland-appearing glands and mild cytologic atypia, making it very difficult to distinguish from normal glandular epithelium. Deep cervical stromal involvement and/or subtle stromal response may be the only morphologic features suggesting a malignant process. Therefore, it is very challenging to diagnose well-differentiated GAS in biopsy, conization or even hysterectomy specimens. Routine screening methods (HPV test and/or cytology) can easily miss GAS due to its HPV negativity and bland cytology.

With the increasing implementation of large-scale HPV vaccination, the proportion of HPV-independent cervical adenocarcinomas including GAS may increase rapidly, making it even more important to recognize and accurately diagnose endocervical GAS in its early stage. In this study, we analyzed 23 cases of GAS to summarize its cytologic characteristics, clinicopathological features, and immunohistochemical stains to emphasize the diagnostic pitfalls of this uncommon cervical adenocarcinoma.

## Methods

### Patient selection

Twenty-three patients diagnosed with GAS or MDA between January 2009 and December 2018 were retrieved at Peking University People’s Hospital (PKUPH), including 15 in-house cases and 8 consultation cases. Forty-nine histologic specimens were available from these patients, including cervical biopsies (*n* = 20), vaginal biopsies (*n* = 3), endometrial curettings (*n* = 2), ureteral biopsies (*n* = 2), pelvic biopsy (*n* = 1), loop electrosurgical excision procedures or cold-knife conization (*n* = 4), and hysterectomy specimens (*n* = 17). Clinical characteristics and follow-up results were also obtained from electronic medical record.

### Cytology examination

Pretreatment cytologic slides were made using either ThinPrep Pap test (TPPT) (Hologic, Bedford, MA) or SurePath Pap test (BD Diagnostics, Franklin Lakes, NJ). All cytologic results were reported according to The Bethesda System (TBS) terminology.

### HPV test

Eleven patients had high-risk HPV test using Hybrid Capture 2 assay (HC2, Digene, Gaithersburg, MD, USA). Greater than or equal to 1 pg/ml HPV DNA was identified as positive.

### Immunohistochemical (IHC) analysis

All IHCs were performed according to manufacturers’ protocols. The following IHCs were performed: p53 (D07, Roche, Roche Benchmark Ultra), p16 (E6H4, Roche, Roche Benchmark Ultra), ER (6F11, Leica, RTU), PR (16, Leica, RTU), HIK1083 (HIK1083, Kanto, 1:20), PAX8 (BC12, Biocare, 1:200), CK7 (EP16, zhongshanjinqiao, 1:100), CK20 (EP23, zhongshanjinqiao, 1:100), MUC6 (MRD220, zhongshanjinqiao,1:200), CEA (31,zhongshanjinqiao,1:200), CA-IX(Poly,zhongshanjinqiao,1:200), CDX-2(EP25,zhongshanjinqiao,1:200), Ki-67(EP5,zhongshanjinqiao,1:200). The immunohistochemical stains were evaluated by two pathologists with consensus. IHC for p16 was interpreted as positive if diffuse and block-like nuclear and cytoplasmic staining. IHC for p53 was interpreted as “mutation-type” expression if ≥75% of tumor cell nuclei were strongly positive or completely negative in the presence of intact internal control. For ER, PR, PAX8, CDX2, and Ki-67, only nuclear staining was considered positive. For CK7, CK20, MUC6, HIK1083, CEA and CA-IX, cytoplasmic staining was read as positive.

## Results

### Clinical features

The study cohort included 23 patients diagnosed with endocervical GAS. The average age was 51.0 years old (range: 28–73 years). The most common symptom was vaginal mucoid discharge (40.0%, 6/15) followed by abnormal vaginal bleeding (33.3%, 5/15), abdominal pain (13.3%, 2/15). Two patients were asymptomatic and identified by physical examination. Seven patients had ultrasound evaluation, and all patients showed cervical enlargement or mass, and 5 also had uterine cavity fluid.

### Cytology results

**Fig. 1:**
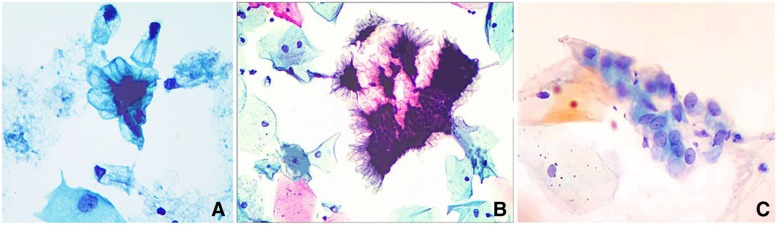
The cytological features of gastric-type adenocarcinoma (GAS). **a** GAS cells display monolayer or honeycomb sheet. The columnar or polygonal tumor cells show pale, foamy, and vacuolated cytoplasm with well-defined cytoplasmic borders. **b** An abundant lacy is present at the periphery of these sheets and the polarity is lost within clusters. **c** The tumor cells are round to oval in shape. Occasional nucleoli can be seen (Papanicolaou staining, 400×)

**Fig. 2:**
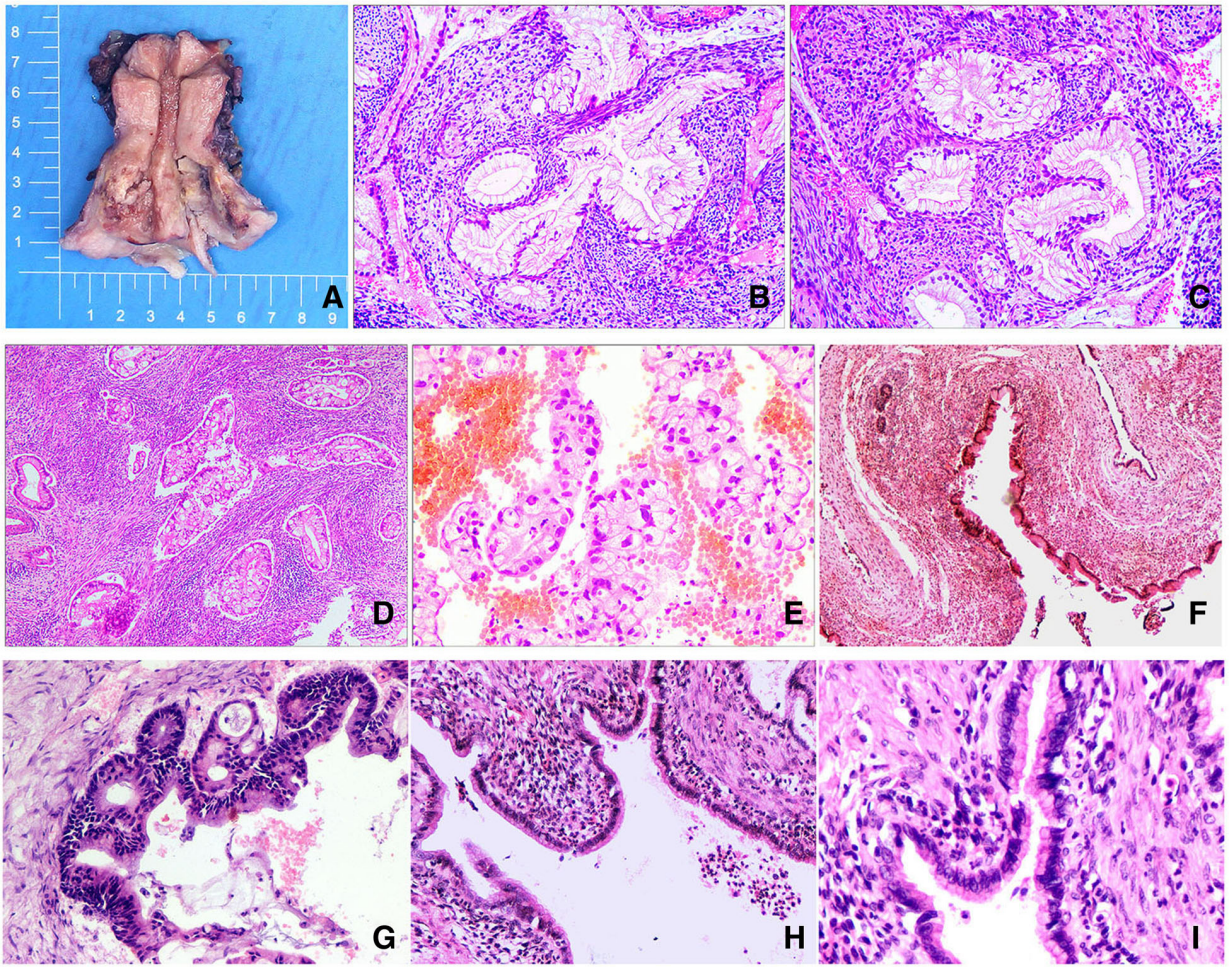
**a** Gross feature of gastric-type adenocarcinoma (GAS). The cervix was partially or entirely replaced by a firm indurated mass imparting a barrel-shaped enlargement. **b**-**c**, Well-differentiated GAS showed haphazardly arranged and irregularly shaped neoplastic glands, which were usually associated with a desmoplastic stromal reaction. The cells showed voluminous, clear to foamy cytoplasm and distinct cellular borders (H&E, 100×). **d** Moderately or poorly differentiated GAS showed extensive epithelial proliferation with crowded, back-to-back glands (H&E, 40×). **e** A GAS showed an area with voluminous clear cytoplasm and round, markedly atypical nuclei (H&E, 100×). **f** Uniform mucinous cells showed minimal or no cytological atypia (H&E, 40×). **g** A GAS case showed mild to moderate nuclear enlargement and pseudostratification (H&E, 200×). **h**-**i**, Bland-appearing mucinous epithelium replaced the fallopian tube normal epithelium and was composed of columnar cells with basally located small nuclei, without distinct nuclear abnormalities (H&E, 100×,200×)

**Fig. 3:**
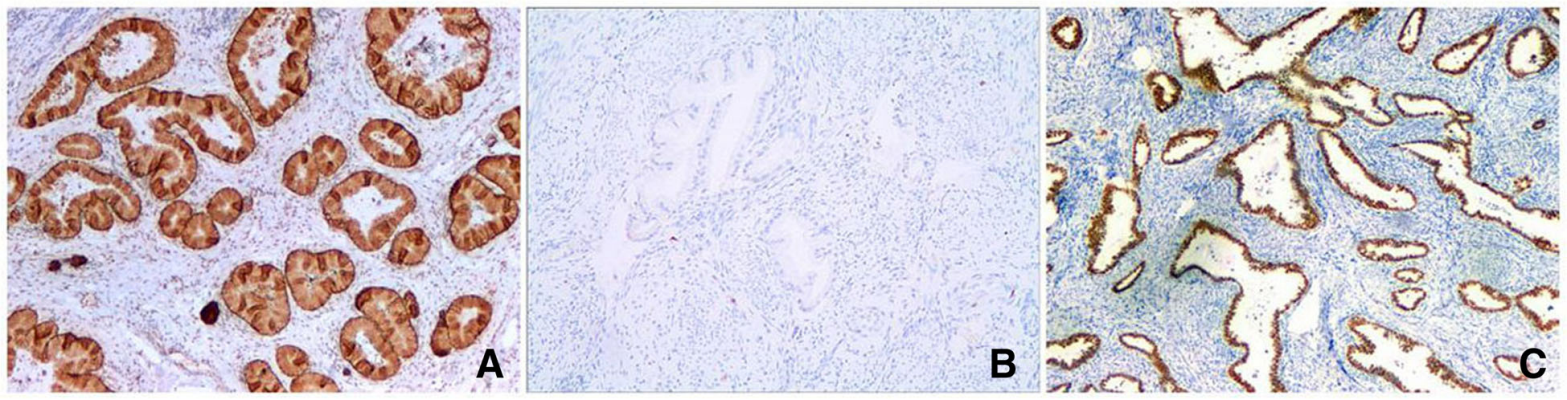
**a** Gastric-type adenocarcinomas (GAS) with diffuse positivity with MUC6. **b** GAS displays negative immunoreactivity with p16. **c** GAS shows diffuse p53 positivity (H&E, 100×)

Eleven patients had previous cervical cytology testing, and the original cytologic interpretations included: Atypical glandular cells, favor adenocarcinoma (AGC, FN) (*n* = 1); Atypical glandular cells, not otherwise specified (AGC,NOS) (*n* = 3); High-grade squamous intraepithelial lesion (HSIL) (*n* = 1); Negative for Intraepithelial Lesion or Malignancy (NILM) (*n* = 5); and Unsatisfactory for evaluation (*n* = 1). Cytologically, the findings included: 1) abundant mucus in the background (Fig. 1 a); 2) numerous glandular cells resembling benign glandular cells arranged in single layers or honeycomb sheets with a palisading pattern with luxuriant lacy at the periphery (Fig. 1 a-b); 3) tall columnar cells with a moderate to abundant amount of mucin in cytoplasm and well-defined cytoplasmic borders (Fig. 1 a-b); 4) slightly enlarged nuclei with finely granular chromatin. Because the cytoplasm contained abundant mucin or vacuoles, the nuclear to cytoplasmic ratio was not increased (Fig. 1 c).

### HPV testing

All HPV tests performed on cytologic specimens from these patients were negative (11/11, 100%).

### Histopathology features

On gross examination, most cases showed a firm, indurated or friable mass, or a “barrel-shaped” enlarged cervix. However, two cases showed exophytic tumors with variably sized cysts. The maximum tumor diameter ranged from 2.5 to 7.0 cm. The cut surface was often white solid or cystic-solid, and areas of hemorrhage and necrosis were seen in some cases (Fig. 2 a).

Fourteen cases with well-differentiated GAS demonstrated very mild cytologic atypia, which could be easily confused with normal glandular epithelium. The cytoplasm was usually pale or eosinophilic and contained mucin. The cell border was usually well-defined and the nuclei were located basally (Fig. 2 b-c). Focal moderate to severe cytologic atypia could be seen. However, neoplastic glands were often angulated, dilated, and arranged haphazardly. The most important diagnostic features included deep stromal invasion of the neoplastic glands and the surrounding stromal reaction (Fig. 2 b-c). The other nine cases were moderately and poorly differentiated, with moderate to severe cytologic atypia, enlarged hyperchromatic nuclei, and occasional prominent nucleoli (Fig. 2 d-e). Mitotic figures were easily identified and cribriform or papillary growth pattern could be seen in these moderately or poorly differentiated cases.

Among 18 cases with pathologic staging, vaginal involvement was present in 7 (38.9%) cases and uterine corpus involvement in 9 (50.0%) cases. Ovary was involved in 5 (27.8%) cases with 2 bilaterally and 3 unilaterally. The largest diameter of ovarian involvement ranged from 2.5 to 8.0 cm. In three cases, metastatic tumors in ovary showed predominantly large dilated cysts or glands. At high magnification, the cysts/glands were lined by single layer of mucinous epithelium with mild atypia and pale/eosinophilic cytoplasm. Stratification and papillary structures were rarely seen. Their morphology strongly mimicked a primary benign or borderline ovarian mucinous tumor (Fig. 2f-g). In the other two cases, ovarian metastatic tumors showed destructive stromal infiltration with atypical mucinous glands.

Fallopian tube involvement was present in 6 cases (3 left, 3 right). Two of them showed mucosal colonization without stromal invasion, one showed stromal infiltration without mucosal involvement; the remaining three cases showed both mucosa and stromal involvement. Most fallopian tube mucosal involvements were in a manner of single layer mucinous epithelium with mild atypia (Fig. 2h). Focal areas with multilayer mucinous epitheliums or papillary architecture were rarely seen (Fig. 2i). Lymphovascular space invasion was identified in 13 (13/18, 72.2%) cases.

### Immunohistochemical study

CK7, CK20, MUC6, CEA, CA-IX, PAX8 and CDX2 staining were performed in most of the cases and were positive in 17 (17/17, 100.0%),4 (4/17, 23.5%), 15 (15/15100.0%), 17 (17/20, 85.0%), 13 (13/15, 86.7%), 9 (9/14, 62.3%) and 4 (3/16, 25.0%) cases, respectively. Staining was typically diffused for CK7, MUC6 (Fig. 3a) and CA-IX, but patchy for CK20 and CDX2. P16 was either completely negative or just focally positive (Fig. 3b). P53 was performed in 21 cases and 57.1% (12/21) of them showed a “mutation-type” staining pattern (Fig. 3c), including 7 cases with diffuse strong positivity and 5 cases with complete negativity. ER and PR were negative in all 18 tested cases. HIK1083 was performed in 3 cases, and 2 of them showed focal positivity. Ki-67 proliferation index ranged from 5 to 60%.

### Follow-up

Follow-up information was available in twelve patients, with the follow-up time ranging from 5 to 108 months. Five patients were alive with no evidence of disease; two patients were alive with disease; one patient had local recurrence twice at 7 and 9 months after surgery; one patient had metastasis to bladder 6 months after surgery; and four patients died of disease at 2, 10, 20 and 24 months after surgery, respectively.

## Discussion

Malignant Adenoma was first described in 1870 by German gynecologist Gusserow [5], and its another terminology, minimal deviation adenocarcinoma, was proposed by Silverberg and Hurt in 1975 to imply its similarity to the normal endocervical gland and the lack of overt malignant cytological feature [6]. In 1998, a Japanese study group demonstrated that MDA showed immunoreactivity for HIK1083 and/or MUC6, suggesting gastric pyloric mucin [4]. Moreover, the concept of cervical mucinous adenocarcinomas of gastric subtype as a distinct entity was proposed by Kojima et al. [4], who defined GAS as a tumor composed of neoplastic cells showing clear and/or pale eosinophilic and voluminous cytoplasm with well-defined cell boundaries. Although MDA and GAS each exhibit unique morphology in its pure pattern, it is now recognized that these belong to one family with mixed morphology. In 2014 WHO Classification of Tumors of the Female Reproductive Organs, GAS and MDA were classified under the same category of “gastric type mucinous carcinoma”, and MDA was referred to an extremely well-differentiated form of GAS [7].

Early study has reported MDA accounts for about 1% of cervical adenocarcinomas, and occurs in patients with a wide range of age from 25 to 72 years old (average: 42 years old) [7]. In recent studies of GAS, the average age of the patients was 49 to 51 years old, similar to the average age of our cohort of GAS patients (51 years old) [4, 8]. The common symptoms in our cohort of GAS patients with GAS include irregular vaginal bleeding and discharge, which are similar to those in patients with other types of cervical adenocarcinomas and lack of specificity.

High-risk HPV DNA testing and Papanicolaou (Pap) smear testing are routinely used for cervical cancer screening. However, HPV test does not play any important role in screening endocervical GAS since it is not caused by HPV infection, leaving cytologic evaluation as the only possible screening method to detect GAS. However, previous study [9] has suggested cytology had a low sensitivity (32.7%) to detect MDA or GAS, which could attribute to the bland cytologic features, and their location being more often in the upper endocervical canal. Some subtle cytologic features were suggested to include monolayer and honeycomb sheets with vesicular nuclei, prominent nucleoli, vacuolar or foamy cytoplasm, and intracytoplasmic neutrophil entrapment [10]. In this study, we also found a majority of glandular cells closely similar to normal endocervical cells arranged in sheets, strips, and isolated cells. An abundant lacy was evident at the periphery of these sheets and the cell polarity was lost within clusters. Three -dimensional cell clusters with enlarged, pleomorphic hyperchromatic nuclei were not observed in our cases. Some researchers also reported that “Golden-yellow intracytoplasmic mucin” was occasionally seen on cytologic slides [10] and thought to be a specific indicator for GAS [11]. But this feature was not noticeable in our cases, probably due to the small sample size in our study.

Histologically, the neoplastic glands in moderately and poorly differentiated GAS are usually variable in size and shape, show extensive and deep invasion into cervical stroma. The tumor cells show clear or pale eosinophilic cytoplasm and well-defined cell boundaries. The nuclei are pleomorphic and hyperchromatic with one or more prominent nucleoli. However, the well-differentiated GAS including MDA often presents with well-form glands with bland columnar cells rich in mucin with basal nuclei showing minimal or slight pleomorphism, although moderate to severe atypia may be seen focally.

Synchronous mucinous metaplasia and neoplasia of the female genital tract (SMMN–FGT), including the cervix, endometrium, ovary, and fallopian tube has been reported before [12, 13]. This lesion shows a unique set of morphological features ranging from metaplasia to adenocarcinoma. Mikami et al. [12] considered SMMN–FGT might be a consequence of lobular endocervical glandular hyperplasia (LEGH) or pyloric gland metaplasia (PGM). Most synchronous ovarian and endocervical mucinous tumors were previously interpreted as SMMN–FGT or independent primary endocervical and ovarian/ fallopian tube neoplasms. [12, 13] In this study, we found 6 cases had fallopian tube involvement, and 5 cases had concurrent ovarian mucinous lesions. Most of them showed benign-appearing mucinous glands infiltrating into fallopian tube and/or ovary, mild-to-moderate degrees of nuclear atypia and focal pseudostratification growth pattern. Given the deeply invasive endocervical GAS and the presence of focal areas of prominent nuclear atypia in fallopian tube or ovarian lesions, the benign-appearing extra-cervical mucinous lesions were considered as metastatic diseases, rather than SMMN–FGT or independent primary tumors. In addition, all of these cases had extensive lymphovascular space invasion, supporting metastatic disease.

As previously suggested, positive staining with MUC6 and/or HIK1083 was one of the main reasons for designating such lesion as GAS [4]. In our study, MUC6 was positive in all cases, higher than Carleton et al. [14] reported MUC6 positive rate (81%), and the positive staining was more often diffuse than focal. HIK1083 is a specific but not highly sensitive marker for GAS in the previous study, which showed 66.7% (2/3) of cases with HIK1083 focal positivity [14]. However, this antibody is not commercially widely available, limiting its clinical use. CA-IX is positive in both HPV-related endocervical adenocarcinoma and GAS [14–16]. In current study, CA-IX was positive in 86.7% of cases, which was similar to previously reported positive rate (83.0%) [14]. P16 was negative or focally positive in 90.0% of cases, and two cases showed diffuse p16 positivity despite negative HPV DNA testing. It has been reported that diffuse p16 expression is not always related to the presence of oncogenic HPV [17]. A recent series reported mutation-type p53 staining in 41% of GAS [14], and suggested that p53 mutation may play a role in the pathogenesis of GAS. In our study, 57.1% of cases showed p53 mutation-type staining, which was similar to the results from Simona et al. (51%) [16]. CK7, CEA and PAX8 are usually positive in GAS, while ER and PR are often negative. Our results for these immunostains are consistent with the previous literature [14, 16, 18].

The differential diagnosis of GAS needs to include both benign and malignant lesions due to its complex and diverse morphologic features. The most common differential diagnosis of well-differentiated GAS includes LEGH and diffuse laminar endocervical glandular hyperplasia (DLEGH). LEGH often shows distinct lobular structures with hyperplasia of small circular glands, which usually surround a central dilated duct. However, a small group of LEGH may show cytologic atypia and/or structural complexity, three markers (SMA, p53 and PAX2) could help in the differential diagnosis in equivocal cases. SMA positivity may highlight the desmoplastic stromal reaction in GAS, but there is no staining of SMA around LEGH [19]. In addition, if present, p53 mutation-type immunohistochemical staining could differentiate GAS from LEGH (wild-type p53 staining). In one study, PAX2 staining was reported to be positive in all cases of LEGH and negative in GAS [20].

The differential diagnosis of moderately and poorly differentiated GAS includes usual-type endocervical adenocarcinoma, intestinal type/ signet ring type mucinous adenocarcinoma and metastatic mucinous adenocarcinoma to cervix (gastrointestinal or pancreatobiliary). Usual-type endocervical adenocarcinoma usually shows less mucinous cytoplasm, moderate to severe pleomorphic nucleus, abundant mitotic figures and apoptotic bodies, and p16 diffuse positivity. Intestinal-type endocervical adenocarcinoma usually shows goblet and/or Paneth cell, whereas GAS displays gastric type differentiation. Immunohistochemically, MUC-6 and/or HIK1083 are usually negative and p16 is diffusely positive in intestinal-type endocervical adenocarcinoma. The clear cytoplasm in GAS may confuse it with clear cell carcinoma (CCC), which usually shows a tubulocystic, papillary, or solid structure. Immunohistochemically, GAS and CCC both show positivity for HNF-1β and NapsinA, but CCC is negative for CA-IX and MUC6, which are more likely positive in GAS [14]. Distinguishing metastatic gastrointestinal or pancreatobiliary adenocarcinomas from primary cervical GAS can be challenging due to morphological similarity and overlapping immunohistochemistry. Knowledge of patient’s prior clinical history will be helpful, whereas LEGH or adenocarcinoma in situ can support cervical primary. PAX8 positivity is in favor of primary cervical GAS, however, since not all cervical GAS cases are PAX8 positive (62.3% in our study), a negative result should not rule out a cervix primary.

## Conclusions

We have described both cytologic and histologic features of GAS and highlighted the distinct features of GAS metastasis to ovaries and fallopian tubes. With large-scale implementation of HPV vaccine, the incidence of HPV-associated cervical adenocarcinoma is expected to decrease. The relative proportion of GAS and other rare HPV negative adenocarcinomas would increase. Awareness of the morphologic features and immunohistochemical profile of GAS will allow pathologists to recognize and accurately diagnose this rare and aggressive entity.

## Data Availability

The datasets generated during and/or analyzed for this study are available from the corresponding author upon reasonable request.

## Abbreviations

CCC: Clear cell carcinoma
DLEGH: Diffuse laminar endocervical glandular hyperplasia
FIGO: International Federation of Gynecology and Obstetrics
GAS: Gastric- type adenocarcinoma
HPVA: Human papillomavirus-associated adenocarcinoma
LEGH: Lobular endocervical glandular hyperplasia
MDA: Minimal-deviation adenocarcinoma
NHPVA: Non human papillomavirus-associated adenocarcinoma
PGM: Pyloric gland metaplasia
SMMN–FGT: Synchronous mucinous metaplasia and neoplasia of the female genital tract
